# Oxidative stress promotes exit from the stem cell state and spontaneous neuronal differentiation

**DOI:** 10.18632/oncotarget.23786

**Published:** 2017-12-30

**Authors:** Qidong Hu, Puja Khanna, Belinda Shu Ee Wong, Zealyn Shi Lin Heng, Charannya Sozheesvari Subhramanyam, Lal Zo Thanga, Sharon Wui Sing Tan, Gyeong Hun Baeg

**Affiliations:** ^1^ Department of Anatomy, Yong Loo Lin School of Medicine, National University of Singapore, MD 10, Singapore

**Keywords:** Ntera2 cell, differenitation, reactive oxygen species, oxidative stress

## Abstract

Reactive oxygen species (ROS) play important roles in fundamental cellular processes such as proliferation and survival. Here we investigated the effect of oxidative stress on stem cell maintenance and neuronal differentiation in a human embryonic stem cell (hESC) model, Ntera2 (NT2). CM-H2DCFDA and DHE assays confirmed that the oxidizing agent paraquat could induce a high level of ROS in NT2 cells. Quantitative PCR, Western blotting and immunocytochemistry showed that paraquat-induced oxidative stress suppressed the expression of stemness markers, including NANOG, OCT4 and TDGF1, whereas it enhanced the spontaneous expression of neuronal differentiation markers such as PAX6, NEUROD1, HOXA1, NCAM, GFRA1 and TUJ1. The treated cells even exhibited a strikingly different morphology from control cells, extending out long neurite-like processes. The neurogenic effect of ROS on stem cell behaviour was confirmed by the observations that the expression of neuronal markers in the paraquat-treated cells was suppressed by an antioxidant while further enhanced by knocking down Nrf2, a key transcription factor associated with antioxidant signaling. Lastly, paraquat dose-dependently activated the neurogenic MAPK-ERK1/2, which can be reversed by the MEK1/2 inhibitor SL327. Our study suggests that excessive intracellular ROS can trigger the exit from stem cell state and promote the neuronal differentiation of hESCs, and that MAPK-ERK1/2 signaling may play a proactive role in the ROS-induced neuronal differentiation of hESCs.

## INTRODUCTION

Reactive oxygen species (ROS), including superoxide (O-), hydroxyl free radicals (HO•) and hydrogen peroxide (H_2_O_2_), are constantly generated as the result of normal cellular metabolism. In mammalian cells, ROS are mainly generated by membrane-bound NADPH oxidase (NOX) complexes, mitochondria and endoplasmic reticulum [1–4]. Low-to-moderate steady-state levels of ROS are known to be essential for cellular proliferation, differentiation, and survival [5]. However, increased intracellular ROS levels can result in oxidative stress, and cause cellular dysfunctions that lead to accelerated ageing and various human diseases [6, 7]. Hence, it is essential to maintain the redox homeostasis by balancing ROS generation and scavenging systems. The Keap1 (Kelch-like ECH-associated protein 1)/Nrf2 (nuclear factor E2-related factor 2) complex serves as a key sensor of oxidative stress. Nrf2 is a transcription factor that controls the expression of a large pool of antioxidant and detoxifying genes in response to oxidative stress [8]. It is negatively regulated by its physical association with the repressor Keap1 in cytoplasm [9]. However, upon stimulation by oxidative stress, Keap1 becomes inactivated, hence allowing Nrf2 to translocate into nucleus. Nrf2 then engages in the transcriptional regulation of many antioxidant and detoxification genes, which are involved in the cellular response to oxidative and electrophilic stress, via antioxidant response element (ARE) [8, 10]. The antioxidant enzymes, such as superoxide dismutase (SOD), catalase, glutathione peroxidase (GPx) and peroxiredoxin (Prx), participate in various enzymatic reactions to convert O- to H_2_O and dioxide (O_2_) [11–13].

Interestingly, a number of studies, most of which focused on hematopoietic stem cells (HSCs), have shown that redox homeostasis is critical in maintaining the self-renewal capacity of mammalian stem cells [12, 14, 15]. For example, low level of ROS in stem cell niches is of importance to maintain the stem cell identity of HSCs [16]. When stem cells are exposed to oxidative stress by the disruption of redox homeostasis, they undergo the process of senescence or apoptosis [17]. Similarly, the differentiation of human adipose tissue-derived multipotent adult stem cells toward a neural phenotype was promoted by an increase in ROS [18]. Furthermore, murine ESCs were also known to maintain their stemness and pluripotency under physiological oxygen levels (2%), but when exposed to prolonged ROS they underwent apoptosis, suggesting the critical role of redox homeostasis in stem cell maintenance [19, 20].

Although redox homeostasis has been implicated in the maintenance of stem cells, the molecular regulators of redox homeostasis within stem cells and the ROS-mediated effectors of stem cell behaviour remain largely unexplored. In addition, only a few studies have examined the function of ROS in hESCs. Therefore, establishing a reliable and easy-to-use cellular model for the functional studies of ROS may help gain valuable insights into how hESC fate is determined by redox homeostasis and lead to the development of strategies to manipulate hESC *ex vivo*. Human embryonal carcinoma (hEC) NT2 cells closely resemble hESCs in terms of their pluripotency. When they are injected into immune-deficient mice, they can form teratoma with three primary germ layers [21]. NT2 cells also highly express master regulators such as NANOG (Nanog homeobox), OCT4 (octamer-binding transcription factor 4), SOX2 (SRY-box 2) and TDGF1 (teratocarcinoma-derived growth factor 1), which were previously identified in hESCs [21, 22]. Hence, they have been extensively used to dissect the differentiation of hESCs, especially the all-trans retinoic acid (atRA)-induced neuronal commitment [23]. In this study, we attempted to assess whether elevated ROS level and subsequent redox signaling can instruct the differentiation of NT2 cells into neuronal cells. We showed that oxidative stress suppresses the expression of stemness genes but conversely enhances the spontaneous expression of neuronal marker genes. Importantly, the increase in neuronal markers was attenuated by the treatment of cells with an antioxidant. Lastly, our study implicated MAPK-ERK1/2 in the ROS-mediated neuronal differentiation of NT2 cells.

## RESULTS

### Treatment of NT2 cells with paraquat causes an increase in ROS level

**Figure 1:**
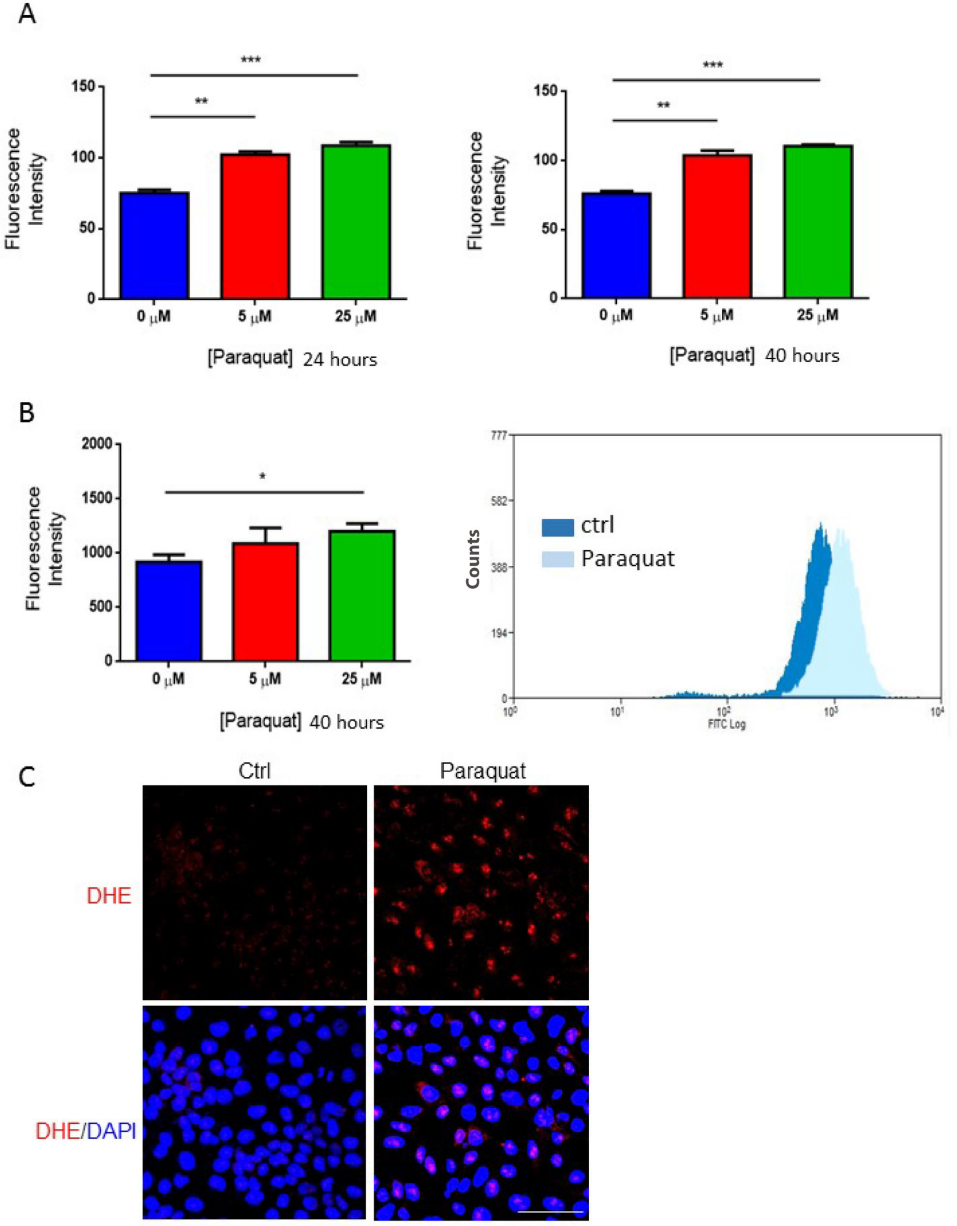
Paraquat increases ROS level in NT2 cells (**A**) Cells were treated with paraquat at the indicated concentrations for 24 hours (left panel) and 40 hours (right panel). Chloromethyl-H2DCFDA dye was used to measure ROS level using spectrometry. Bar: mean ± SEM; ^**^p < 0.01, ^***^p < 0.001. (**B**) Cells were treated with paraquat for 40 hours. ROS level was assessed by staining with chloromethyl-H2DCFDA, and fluorescence intensity was monitored by flow cytometry. Bar: mean ± SEM; ^*^p < 0.05. (**C**) Cells treated with 25 μM paraquat were stained with dihydroethidium (DHE) to visualize the induction of ROS. Scale bar: 100 μm.

To elucidate the potential role of redox homeostasis in the fate selection of hESCs, we first examined whether the oxidizing agent paraquat can induce oxidative stress in the model cell line NT2 cells. The cells were treated with paraquat at various concentrations for 24 and 40 hours, respectively, and an intracellular ROS level was measured using chloromethyl-H2DCFDA fluorescent probe. At both the time points, the addition of paraquat induced a dose-dependent increase in ROS level (Figure 1A). This was further confirmed by monitoring ROS level using flow cytometry (Figure 1B). DHE is a specific fluorescent probe that detects superoxide radicals. Once oxidized it is intercalated into DNA and generates a red fluorescence. In paraquat-treated NT2 cells, most of them showed red staining in their nuclei, whereas non-treated cells were mostly unlabeled (Figure 1C). All these findings suggest that treatment with oxidizing agents induce oxidative stress in NT2 cells.

### A high level of ROS decreases the expression of stemness-related genes

**Figure 2:**
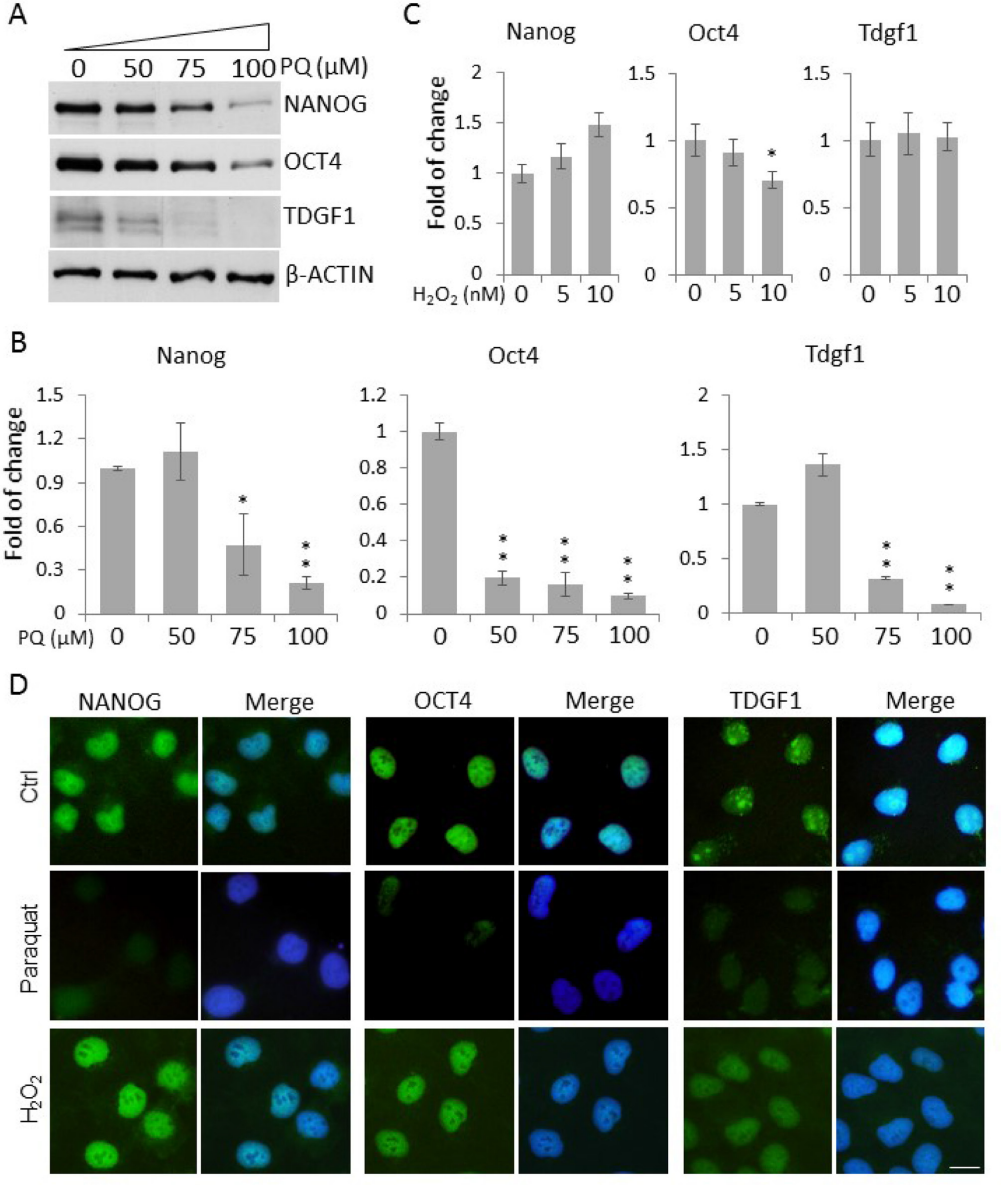
Enhanced ROS decrease the expression of stemness genes in NT2 cells (**A**) Cells treated with paraquat (PQ) at the indicated concentrations were lyzed, and the protein levels of stemness markers, NANOG, OCT4 and TDGF1, were examined by Western blot. β-ACTIN was used as the loading control. Cells treated with indicated concentrations of PQ (**B**) or H_2_O_2_ (**C**) for two days were lyzed for total RNAs. The transcript levels of stemness genes were measured by qPCR. Bar: mean ± SD; ^*^p < 0.05, ^**^p < 0.01 by Student *t*-test. (**D**) Cells were treated with 50 μM paraquat or 10 nM H_2_O_2_ for six days and immunostained for the above stemness markers. The cells were counterstained with DAPI. Scale bar: 20 μm.

A low level of ROS has been shown to be essential to maintain the stemness and pluripotency of mammalian ESCs [19, 20]. This prompted us to test the hypothesis that an elevated level of ROS conversely promotes the exit of NT2 cells from the stem cell state. We first examined the expression of classic ESC markers, including NANOG, OCT4 and TDGF1, in NT2 cells. As expected, Western blotting showed an abundant expression of those stemness markers in control NT2 cells. However, paraquat dose-dependently reduced their protein levels, especially at the higher concentrations tested, 75 μM and 100 μM (Figure 2A). This dose-dependent effect of paraquat on stemness gene expression was further confirmed by quantitative PCR (qPCR) in which higher doses of paraquat (75 and 100 µM) almost completely suppressed the mRNA expression (Figure 2B). However, when cells were treated with another oxidizing agent, hydrogen peroxide (H_2_O_2_), the transcript levels of stemness factors remained largely unaltered as determined by qPCR, except Oct4 showing a moderate decrease in cells exposed to a higher dose of H_2_O_2_ (Figure 2C).

Consistently, immunocytochemical analysis showed a dramatic decrease in the fluorescence intensity of these stemness markers in paraquat-treated cells (Figure 2D), suggesting that NT2 cells under oxidative stress lose their stemness. In alignment with the qPCR data, H_2_O_2_ treatment did not lead to a pronounced decrease in the immunolabeling of NANOG, OCT4 or TDGF1 (Figure 2D), suggesting that it is less potent than paraquat in reducing stemness.

### A high level of ROS enhances the expression of neuronal differentiation markers

**Figure 3:**
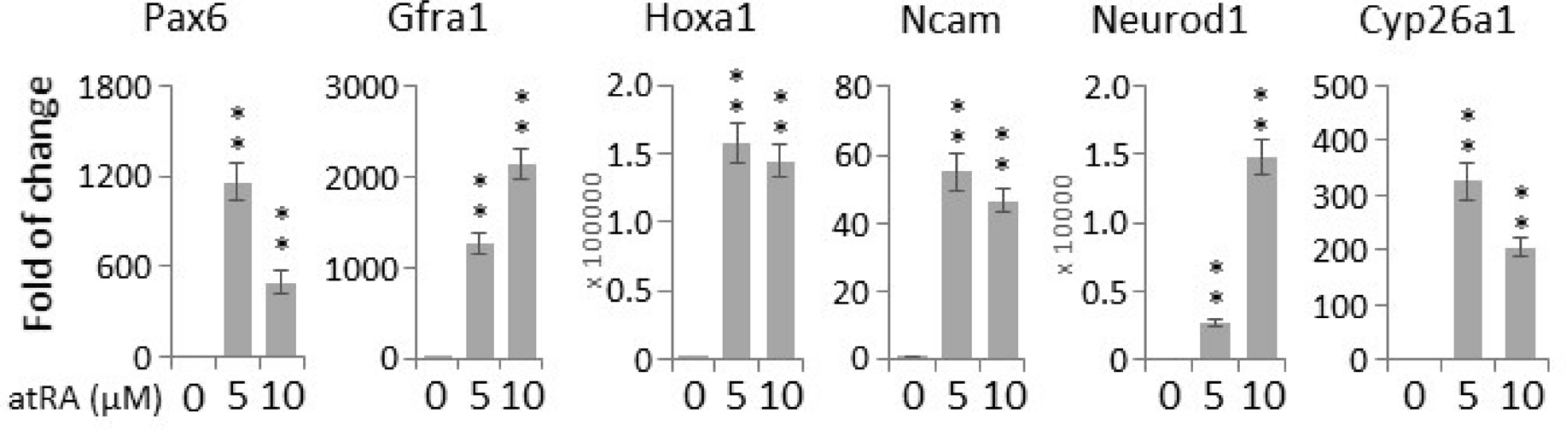
All-trans retinoic acid (atRA) induces neurogenic gene expression in NT2 cells Cells were treated with indicated concentrations of atRA for two days, and the transcript levels of canonical neuronal genes were determined by qPCR. Bar: mean ± SD; ^**^p < 0.01 by Student *t*-test.

**Figure 4:**
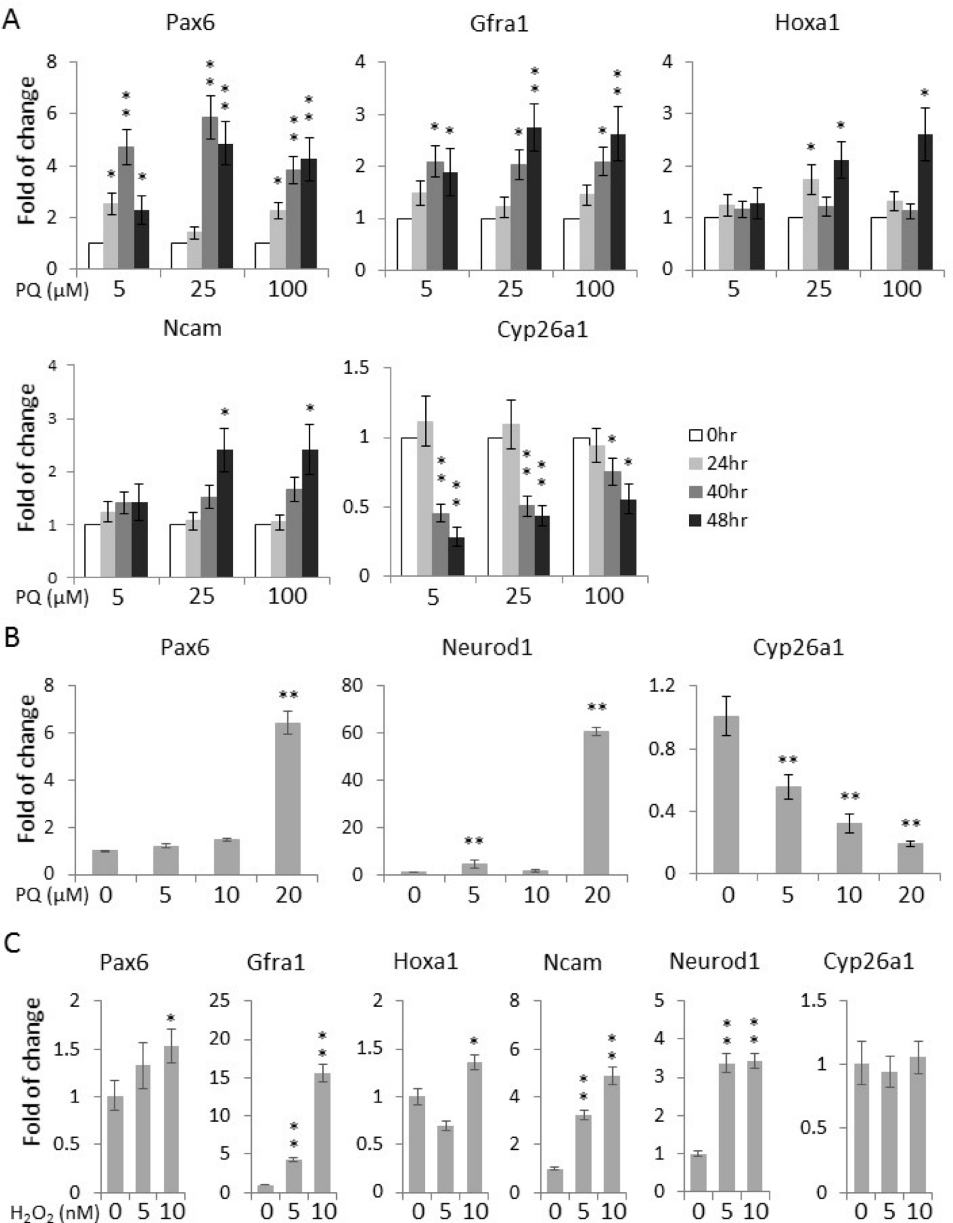
Paraquat induces the expression of neuronal markers in NT2 cells (**A**) Cells were treated with paraquat for different durations. qPCR was performed to measure the time-dependent expression of neuronal markers. (**B**) Cells were treated with indicated concentrations of paraquat for two days. The dose-dependent expression of neuronal transcription factors (*Pax6* and *NeuroD1*) and *Cyp26a1* was quantified by qPCR. (**C**) Cells were treated with H_2_O_2_ at different concentrations for two days and analysed by qPCR for the transcript levels of neuronal marker genes. Bar: mean ± SD; ^*^p < 0.05, ^**^p < 0.01 by Student *t*-test.

The differentiation of human adipose tissue-derived multipotent adult stem cells towards a neural phenotype was shown to be accompanied by an increase in ROS levels [18]. Hence, we proceeded to examine whether the paraquat-induced elevation of ROS levels can spontaneously initiate the neuronal differentiation of NT2 cells. First, to verify the neurogenic potential of NT2 cells, we treated the cells with the well-established neurogenic agent, atRA [24]. qPCR showed that atRA could potently induce the expression of a panel of neurogenic genes, including *Pax6* (Paired box 6) [25], *Gfra1* (GDNF family receptor alpha 1) [26], *Hoxa1* (Homeobox A1) [27], *Ncam* (Neural cell adhesion molecule 1) [28] and *Neurod1* (Neuronal differentiation 1) [29], as well as *Cyp26a1* (Cytochrome P450 family 26 subfamily A member 1), an important feedback factor of atRA signaling in both hESCs and NT2 [30, 31] (Figure 3). For comparison, the cells were then treated with paraquat for different durations. Remarkably, at all the three concentrations tested, 5 μM, 25 μM and 100 μM, the expression of these canonical differentiation markers was increased in a time-dependent manners (Figure 4A), suggesting that oxidative stress alone can initiate the neuronal differentiation of NT2 cells. Surprisingly, in response to paraquat, we noticed a time-dependent reduction in the transcript level of *Cyp26a1* (Figure 4A). Since CYP26A1 is a negative regulator of the neurogenic atRA signaling cascade [30, 31], its suppression may render a more permissive environment for the ROS-induced neuronal differentiation of NT2 cells. To further confirm the notion that elevated ROS levels promote neuronal differentiation, we treated NT2 cells with paraquat at different concentrations for 2 days and examined the transcript levels of two neurogenic transcription factors, *Pax6* and *Neurod1*. We found that 20 μM paraquat is sufficient to elicit a significant induction of these factors (Figure 4B). Consistently, we also observed a dose-dependent decrease in *Cyp26a1* expression by paraquat treatment (Figure 4B), suggesting a potential cross-talk between oxidative stress signaling and neurogenic atRA signaling.

Intriguingly, although H_2_O_2_ did not suppress the expression of stemness genes as efficiently as paraquat (Figure 2C, 2D), it could significantly elevate the transcript levels of neuronal genes in a dose-dependent manner (Figure 4C), implying that increased ROS levels could play an important role in maintaining a delicate balance between stemness and differentiation.

### High ROS induce the outgrowth of neurite-like processes in NT2 cells

**Figure 5:**
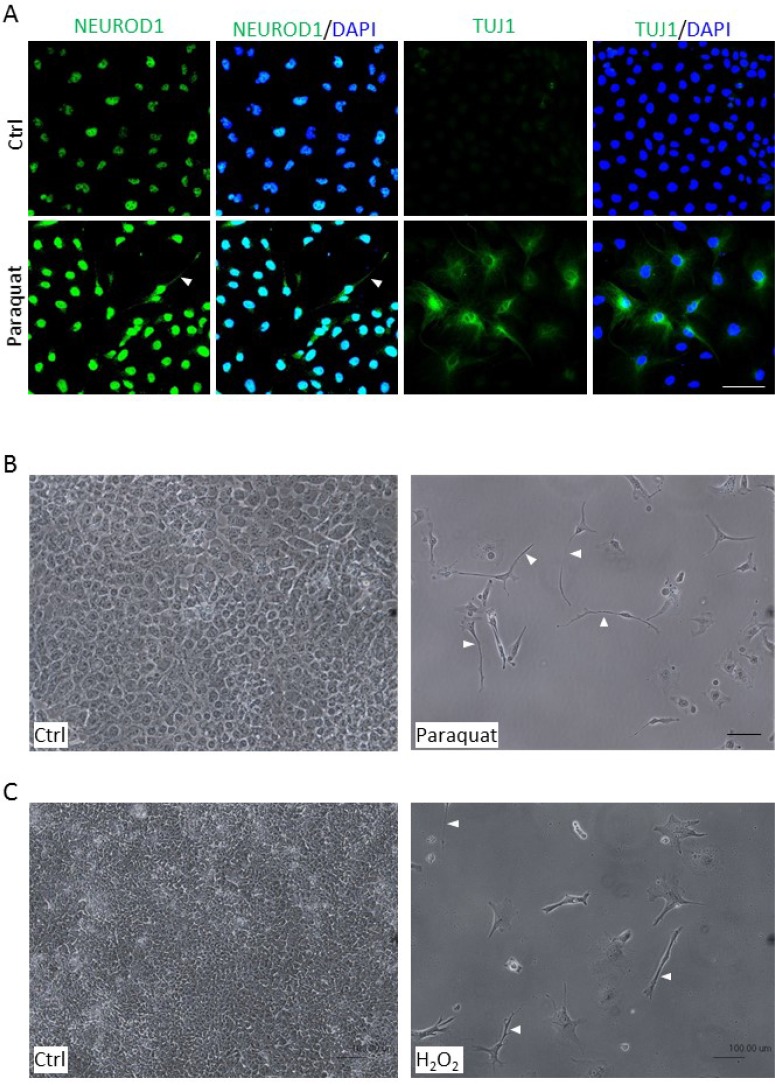
Enhanced ROS cause morphological changes in NT2 cells (**A**) Cells were treated with 25 μM paraquat for six days and immunostained with neuronal markers, NEUROD1 and TUJ1. The cells were counterstained with DAPI. The arrowhead denotes neurite-like cellular process. (**B, C**) Cells were treated with 100 μM paraquat (**B**) or 5 nM H_2_O_2_ (**C**) for six days, and the morphological changes were visualized by phase-contrast microscopy. The arrowheads point to elongated cellular processes. Scale bar: 100 μm.

One of the striking features of neuronal differentiation is the neurite outgrowth, a projection of axons and dendrites [26, 32]. Since paraquat treatment enhanced the expression of neuronal markers (Figure 4A, 4B), we further assessed whether oxidative stress can induce a morphological change in NT2 cells. After treatment with 25 μM paraquat for six days, the cells were immunostained for NEUROD1 and TUJ1 (Tubulin beta 3 class III), a neuron-specific tubulin protein [33]. Consistent with the notion that oxidative stress enhances neuronal differentiation, a significant increase in the staining of NEUROD1 and TUJ1 was observed in treated cells. Importantly, some of the treated cells exhibited an expanded cytoplasm and started to extend out cell processes (Figure 5A, arrowhead), indicating that paraquat-induced expression of neuronal markers can be accompanied by alterations in cell morphology. To further relate the observed morphological changes to neurite-like outgrowth, we treated NT2 cells with the higher concentration of paraquat at 100 μM for six days. As expected, the treated cells showed a much lower proliferative activity as compared to control cells, and exhibited a striking bipolar extension of cell processes which is reminiscent of the neurite outgrowth (Figure 5B, arrowhead). To examine whether other reactive oxygen species could also induce the neurogenic morphological change, the cells were exposed to 5 nM H_2_O_2_. Remarkably, we observed a significant decrease in cell proliferation but a prominent outgrowth of cell processes (Figure 5C, arrowhead), suggesting that oxidative stress can generally induce neuronal differentiation.

### Antioxidant treatment impedes high ROS-mediated neuronal differentiation

**Figure 6:**
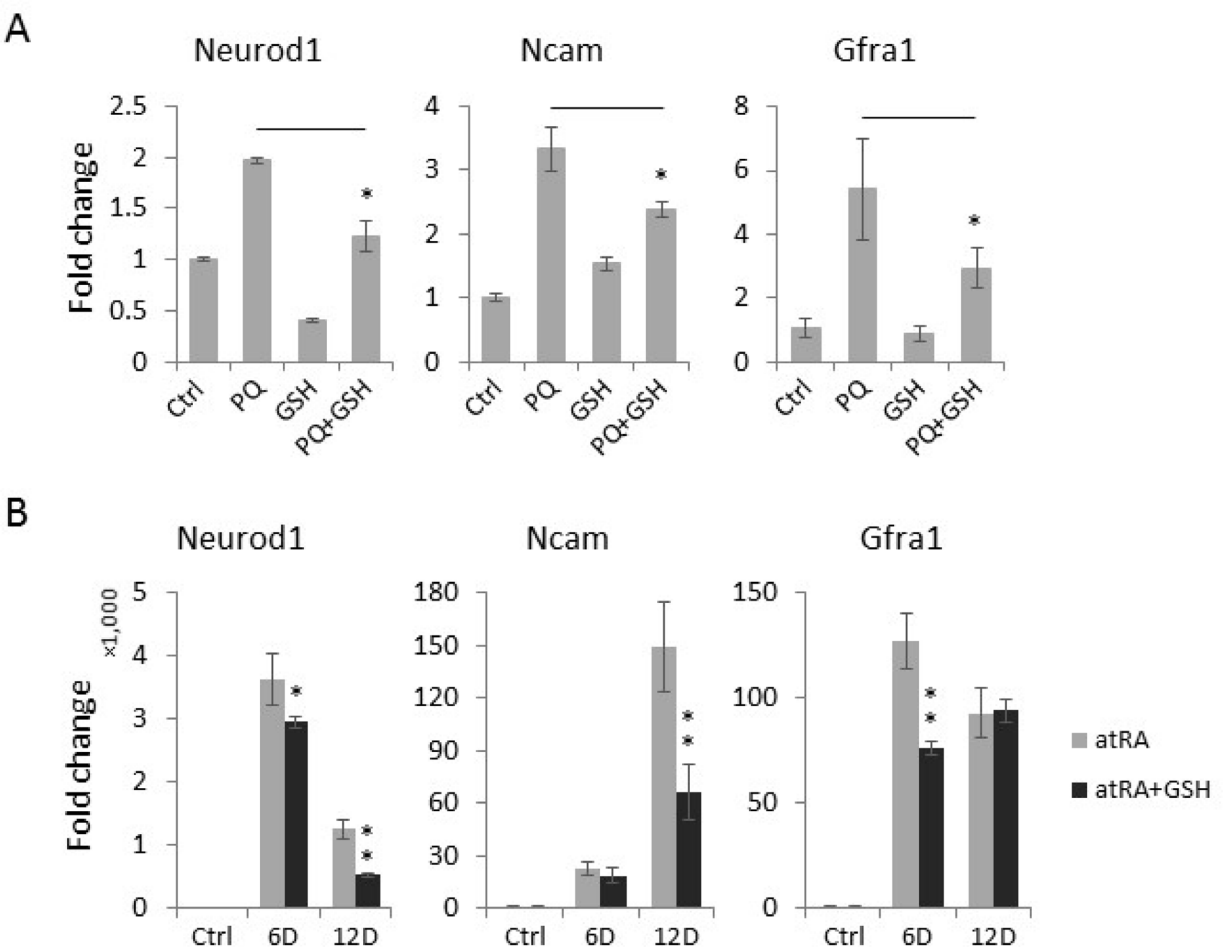
The antioxidant GSH suppresses the expression of neuronal markers induced by paraquat (PQ) (**A**) NT2 cells treated as indicated were collected for total RNA extraction. The expression of neuronal markers was measured by qPCR. (**B**) Cells were treated with atRA (RA) or co-treated with GSH for indicated days (D). The expression of neuronal markers was measured by qPCR. Bar: mean ± SD; ^*^p < 0.05, ^**^p < 0.01.

To confirm that the paraquat-induced spontaneous expression of neuronal genes was mediated by oxidative stress, we assessed whether the concurrent treatment of NT2 cells with the antioxidant glutathione (GSH) can antagonize the neurogenic effect of paraquat. Interestingly, we observed that GSH significantly reduces the paraquat-induced expression of neuronal differentiation markers, including *Neurod1*, *Ncam* and *Gfra1* (Figure 6A). To test whether an elevated level of ROS is a common cause of neuronal differentiation in stem cells, we next examined the effect of GSH on atRA-mediated neuronal differentiation by co-treating NT2 cells with GSH and atRA, a well-established neurogenic agent. Remarkably, the addition of GSH in part attenuated the effects of atRA on the expression of neuronal makers (Figure 6B), suggesting that ROS also play important roles in atRA-induced neuronal differentiation of NT2 cells.

### Paraquat-induced ROS influence antioxidant signaling

**Figure 7:**
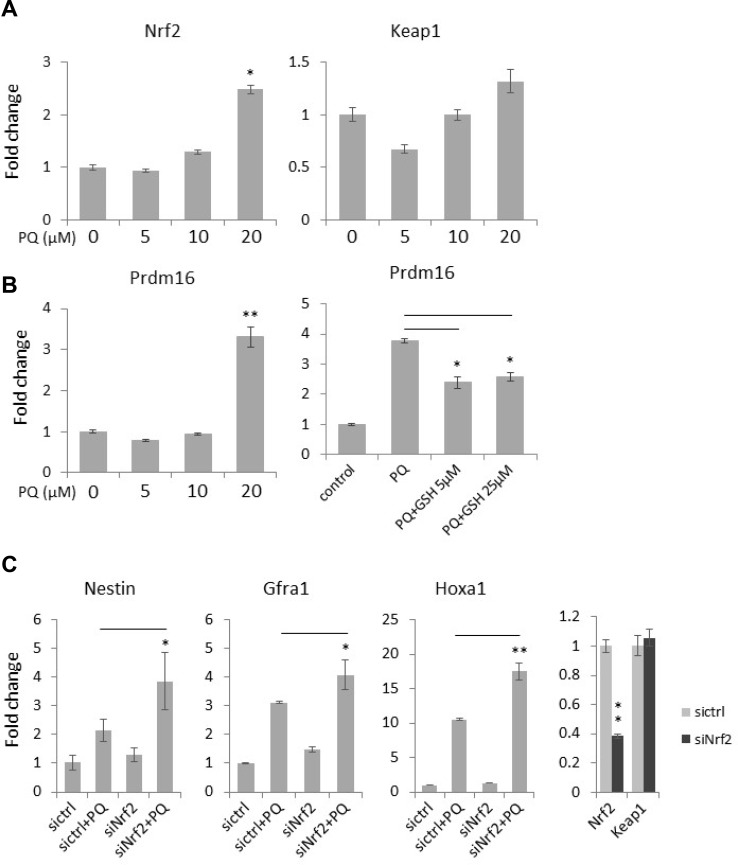
Antioxidant signaling is activated by ROS and is involved in neuronal gene expression in NT2 cells (**A**) Cells were treated with the increasing concentrations of paraquat (PQ) for two days. The expression of the key redox signaling genes, *Nrf2* and *Keap1*, was measured by qPCR. (**B**) Cells were treated with paraquat alone (left panel) or co-treated with paraquat and GSH (right panel). The expression of the antioxidant *Prdm16* mRNA level was examined by qPCR. (**C**) Cells were transfected with siRNA targeting *Nrf2* (siNrf2) and then treated with paraquat for two days. The expression of neural progenitor marker (*Nestin*) and neuronal markers (*Grfa1* and *Hoxa1*) was monitored by qPCR. Bar: mean ± SD; ^*^p < 0.05, ^**^p < 0.01.

The Keap1/Nrf2 pathway plays a major role in maintaining the redox homeostasis. In a physiological condition, Keap1 acts as a cytoplasmic repressor of Nrf2 by binding and promoting its degradation [8, 9]. However, upon oxidative stress, Nrf2 is released from Keap1, translocates into nucleus and engages in the transcriptional activation of various antioxidant and detoxifying genes. In support of this, we found that paraquat treatment increases the expression of *Nrf2* but had no significant effect on *Keap1* expression in NT2 cells (Figure 7A), suggesting a feedback loop to regulate redox balance. PRDM16 (PR domain 16) is also an important antioxidant protein that functions in regulating an intracellular ROS level and thus maintains redox homeostasis [34]. Importantly, it has been shown that the addition of the antioxidant N-acetyl-cysteine to *Prdm16*-deficient mice partially rescues defects in neural stem cell function, suggesting that PRDM16 facilitates neural stem cell maintenance by modulating oxidative stress [34]. With qPCR, we observed a dose-dependent increase in *Prdm16* expression by paraquat treatment similar to that in *Nrf2* (Figure 7A). However, co-treatment of the cells with GSH significantly decreased *Prdm16* expression induced by paraquat, suggesting an intricate PRDM16-modulated feedback loop to regulate redox homeostasis.

Since elevated ROS promote neuronal differentiation, we next examined whether the disruption of the antioxidative Nrf2 pathway can further enhance the expression of neuronal markers induced by paraquat. As expected, the knockdown of *Nrf2* in paraquat-treated cells further enhanced the mRNA levels of *Nestin*, a neural progenitor cell marker [35, 36], *Gfra1* and *Hoxa1* as compared to that in cells treated with paraquat alone (Figure 7B), confirming that a high ROS level facilitates neuronal differentiation via inducing oxidative stress.

### MAPK-ERK1/2 is dose-dependently activated by paraquat

**Figure 8:**
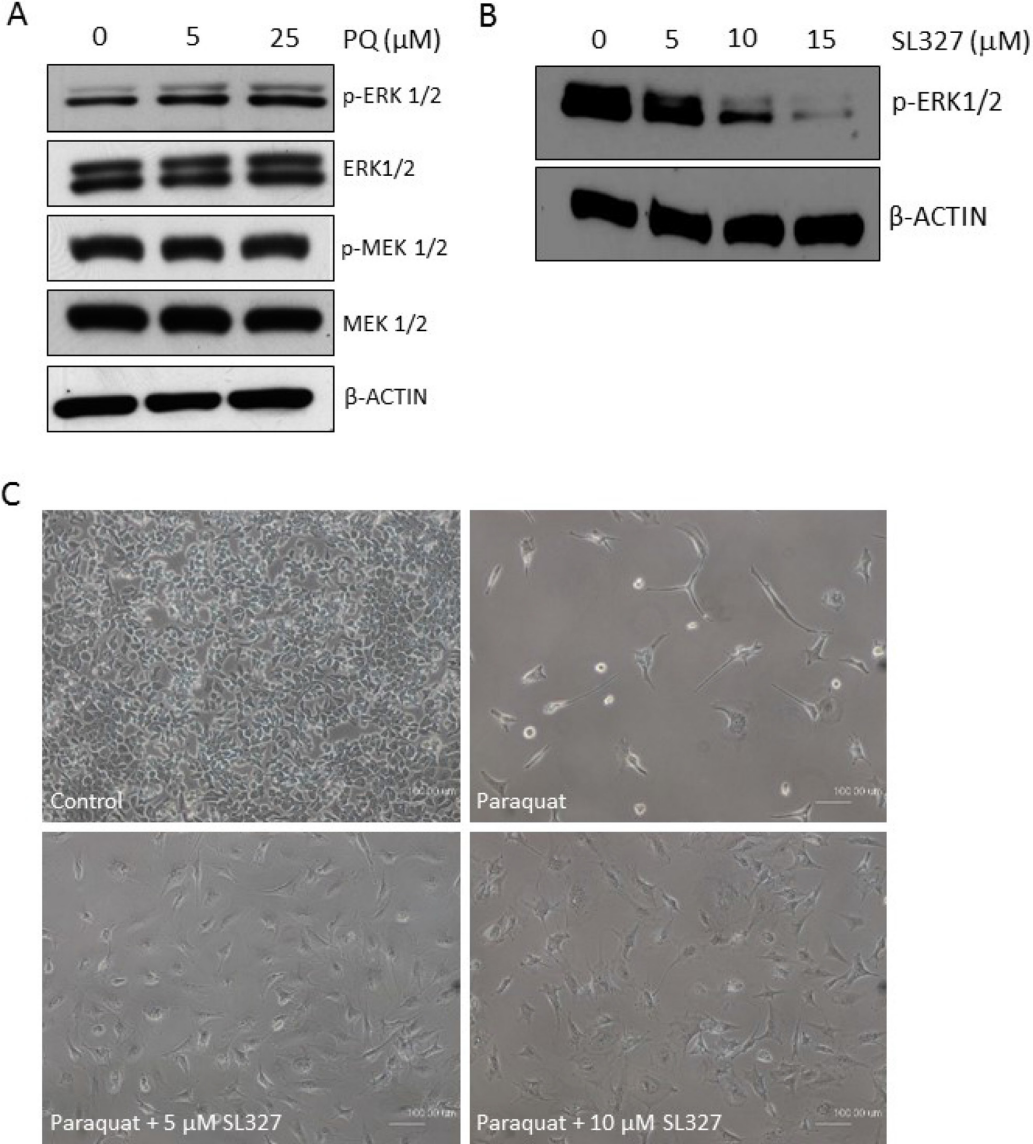
MAPK-ERK1/2 signaling is activated by high ROS in NT2 cells (**A**) Cells were treated with the increasing concentrations of paraquat (PQ). The activation status of ERK1/2 and the upstream MEK1/2 was determined by immunoblotting with indicated antibodies. β-ACTIN was used as the loading control. (**B**) Cells were treated with indicated doses of MEK1/2 inhibitor, SL327. The efficacy of the inhibitor was demonstrated by immunoblotting for phospho-ERK1/2. (**C**) Cells were concurrently treated with 100 μM paraquat and indicated doses of SL327 for 6 days. The phase contrast images showed the cell density and morphology under different treatments. Scale bar: 100 µm.

The mitogen-activated protein kinase (MAPK) cascade is an important signaling pathway that has a major role in multiple biological processes such as development, cell proliferation, apoptosis and stress response [37]. There are three main MAPK subfamilies, ERK1/2, JNK and p38. Notably, a number of studies have implicated the activation of MAPKs in the self-renewal and differentiation of neural stem/progenitor cells or ESCs [38–42]. In particular, atRA treatment has been shown to promote phosphorylation of ERK1/2 in murine ESCs [43]. Hence, we examined whether ERK1/2 become activated in the oxidative stress-mediated neuronal differentiation of NT2 cells. Interestingly, immunoblotting showed that phospho-ERK1/2 level increased in a dose-dependent manner in response to paraquat (Figure 8A). However, MEK1/2, upstream kinases of ERK1/2, were not affected by paraquat. Hence, to confirm the essential role of ERK1/2 in paraquat-induced neuronal differentiation, the cells were concurrently treated with paraquat and the specific MEK1/2 inhibitor SL327 [44], which was shown to decrease phospho-ERK1/2 levels very efficiently in NT2 cells (Figure 8B). The phase contrast images showed that the inhibition of ERK1/2 activation by SL327 dramatically restored the paraquat-suppressed cell proliferation and prevented the neurite-like outgrowth from the cells (Figure 8C). Altogether, these observations suggest that paraquat-induced oxidative stress may promote neuronal differentiation of NT2 cells by activating the MAPK-ERK1/2 signaling pathway.

## DISCUSSION

During the cellular metabolism of oxygen, ROS are constantly produced from NOX complexes in the cell membranes, mitochondria and endoplasmic reticulum [1–4]. However, redox homeostasis is achieved by a delicate balance between the production and the destruction of ROS to ensure the functional integrity of cells, including proliferation, differentiation and survival [5]. A significant deviation from the homeostasis may initiate oxidative stress in cells, resulting in physiological changes and pathological features that include accelerated ageing, neurodegeneration and tumorigenesis [6, 7]. Hence, it is critical to explore the effect of oxidative stress in different cellular contexts, and to characterize the molecular mechanism underlying the physiological and pathological changes.

In this study, we used the human EC cell line NT2 as a surrogate to investigate the role of redox homeostasis in regulating the cell fate of hESCs. It has been shown that a low level of ROS is required for the maintenance of stemness in other stem cell models such as murine ESCs, human HSCs and adipose tissue-derived multipotent adult stem cells [16, 18–20]. Consistently, in NT2 cells, a significant decrease in the expression of some master regulators of stemness, including NANOG, OCT4 and TDGF1, was observed upon oxidative stress induced by paraquat (Figure 2), suggesting that low levels of ROS may be required for hESCs to maintain their stem identity. Our study also provided evidence that paraquat-treated NT2 cells spontaneously express neurogenic transcription factors and neuron-specific structural molecules, including PAX6, NEUROD1, HOXA1, NCAM and TUJ1 (Figure 4A, 4B, 5A). In addition, we observed significant changes in the morphology of the cells, extending out neurite-like cell processes (Figure 5B). These findings are consistent with the previous report, showing that adipose tissue-derived adult stem cells exhibit a neural phenotype when experiencing an increase in ROS level [18]. In fact, our preliminary study using the fruit fly *Drosophila* also showed that redox homeostasis plays important roles in regulating the behaviour of neural stem cells in the developing larval brain (unpublished data).

Surprisingly, the addition of the antioxidant GSH significantly suppressed the expression of neuronal genes induced by the well-known neurogenic agent atRA [33] (Figure 6B), suggesting that redox signaling may be involved in the atRA-induced neuronal differentiation program in NT2 cells. This observation resonates with a recent study that reported the effect of another antioxidant, N-acetyl cysteine (NAC), on neuronal differentiation in human adipose tissue-derived multipotent adult stem cells [18]. Hence, it certainly warrants future studies to characterize the relationship between redox and atRA signaling in the context of neurogenesis. In addition, we found that phospho-ERK1/2 levels are increased upon paraquat-induced oxidative stress in NT2 cells (Figure 8A). Interestingly, the activation status of MEK1/2, an ERK1/2 upstream kinase, was not affected by paraquat treatment, suggesting that ROS signaling may function the downstream of MEK1/2. Nonetheless, as ERK1/2 has been demonstrated to promote neuronal differentiation in murine ESCs [43], our observation supports the finding that oxidative stress facilitates a neuronal fate selection in stem cells. It will be intriguing to further investigate the dynamic crosstalk between redox signaling, MAPK-ERK1/2 signaling and retinoic acid signaling. This will provide us novel and exciting insights into how to promote the exit from stem cell state and to enhance neuronal differentiation in response to various stimuli. This is of particular significance given that many neurodegenerative diseases, including Alzheimer’s disease, Parkinson’s and Huntington’s disease, are characterized by the loss of neurons in the central nervous system. Hence, a comprehensive understanding of the role of ROS in regulating stem cell fate selection may ultimately lead to innovative approaches of generating functional neurons from stem cells to alleviate neurodegeneration.

Besides the nervous system, an accumulation of ROS is closely associated with pathophysiological mechanisms underlying cancer and metastasis [45–49]. Unlike tumour cells, cancer stem cells (CSCs) maintain a low ROS level, although the underlying mechanism remains to be addressed. Many lines of evidence have suggested that CSCs are one of the primary causes for drug resistance and tumour recurrence [50]. Since CSCs have been found to share some basic properties with normal stem cells, including self-renewal and multipotent differentiation capacity, our study on the role of ROS in regulating hEC cell behaviour may also shed light onto some interesting questions, such as how to manipulate ROS level and/or antioxidant machinery in CSCs to induce cell death and to prevent tumour relapse. Indeed, some of oxidizing agents such as niclosamide have been shown to selectively eliminate CSCs of acute and chronic myeloid leukemia [50–53], suggesting that the induction of oxidative stress may cause CSCs to lose stemness, leading to an improved long-term therapeutic outcome.

In summary, future work is urgently needed to elucidate the redox regulatory mechanisms in hESCs which may ultimately lead to new strategies of producing functional neurons to treat neurodegenerative diseases and could be even applied to manipulate the fate of CSCs to improve relapse-free survival.

## CONCLUSIONS

In this study, we successfully induced the oxidative stress in the human EC line NT2 by treating paraquat at various concentrations. We showed that in the treated cells, stemness-related gene expression was downregulated but the expression of neuronal markers was conversely upregulated. Importantly, increased neuronal marker expression in treated NT2 cells was accompanied by a morphological change, which is reminiscent of the neurite outgrowth. Interestingly, the addition of the antioxidant agent GSH reversed the expression of neuronal genes induced by either oxidative stress or atRA. This suggests that atRA-mediated neuronal differentiation also involves redox signaling and that antioxidant activity is required for stem cells to maintain their stem identity. Lastly, we found that oxidative stress activates MAPK-ERK1/2, suggesting a functional crosstalk between oxidative stress signaling and MAPK signaling in the aspect of neurogenesis program in NT2 cells.

## MATERIALS AND METHODS

### Culture of NT2 cells

NT2 cell line was purchased from ATCC (CRL-1973). The cells were maintained in DMEM-GlutaMax™ (#10569010, ThermoFisher Scientific) supplemented with 10% FBS (Hyclone). To induce oxidative stress, the cells were treated with the various concentrations of paraquat (Sigma-Aldrich) or H_2_O_2_ (Sigma-Aldrich) for indicated time periods. The cells were then subjected to various assays. To induce neuronal differentiation, NT2 cells were also treated with 10 µM all-trans retinoic acid (atRA) (Sigma-Aldrich) for indicated time periods. To investigate the effect of antioxidant on neuronal gene expression, oxidized cells were treated with 50 µM glutathione (GSH) (Sigma-Aldrich) for two days. NT2 cells were treated with SL327 (Sigma-Aldrich) to examine the effect of inhibiting MEK1/2 on paraquat-induced neuronal differentiation of the cells. Cells were treated with SL327 at various concentrations for 24 hours (Western blot analysis) and 6 days (phase contrast images), respectively.

NT2 cells seeded in 6-well plates were transfected with Lipofectamine 3000 (Invitrogen) as instructed. The siRNA pool targeting human NRF2 was purchased from Sigma-Aldrich (MISSION^®^ esiRNAs #EHU093471).

### Measurement of ROS levels in NT2 cells

NT2 cells were cultured in 6-well plates up to 80% confluency and treated with 0 µM, 5 µM and 25 µM of paraquat. Cells were then harvested after 24 and 40 hours, and stained with 10 µM chloromethyl-H2DCFDA dye (#C6827, ThermoFisher Scientific) for 30 minutes at 37°C. Intensity of the dye that can reflect ROS levels in the cells was measured using SpectraMax M5 and DAKO CyAn ADP Flow Cytometer, respectively. Statistical analysis was performed on GraphPad Prism 6.0 software. All values were expressed in mean ± standard error of mean. Using unpaired *t*-test, data was analysed and *p < 0.05* was considered statistically significant.

### Immunocytochemistry

NT2 cells were cultured on 13 mm cover slips up to 80% confluency, and then treated with 0 µM and 50 µM of paraquat. Following the treatment, the cells were fixed in 4% paraformaldehyde and permeabilized using 0.2% Triton X-100 in 1X PBS. The cells were blocked in 1% (w/v) BSA in 1X PBS and stained for overnight at 4°C with the following primary antibodies: OCT4 (1:400, #2750, Cell Signaling Technology; CST), NANOG(1:200, #3580, CST), TDGF1(1:200, #2020, CST), β3-tubulin (1:200, #4466, CST), and NeuroD1 (1:100, #4373, CST). The cells were subsequently stained with the following secondary antibodies: DyLight 488-AffiniPure Rabbit Anti-Mouse IgG (H+L) (1:200, Jackson ImmunoResearch Laboratories) and Goat Anti-Rabbit IgG (H+L) Alexa Fluor 488 Conjugate (1:200, #A11008, ThermoFisher Scientific). To measure ROS activity (superoxide level), NT2 cells were stained with 30 µM dihydroethidium (DHE) (#D1168, ThermoFisher Scientific) for 5 minutes. Slides were mounted using Vectashield mounting medium containing DAPI on a glass slide and viewed under the confocal microscope (Olympus Fluoview FV1000 cLSM).

### Protein extraction and Western blot analysis

NT2 cells were cultured up to 80% confluency and treated with 0 µM, 50 µM, 75 µM and 100 µM of paraquat for 40 hours. Protein was extracted using M-PER™ Mammalian Protein Extraction Reagent (#78501, ThermoFisher Scientific) supplemented with Halt™ Protease Inhibitor Cocktail and EDTA (#87786, ThermoFisher Scientific). 30 μg of protein lysate was resolved on 8% SDS-PAGE gels, transferred onto nitrocellulose membrane (Bio-Rad) and then blocked using 5% (w/v) BSA or milk in 1X TBST. The membrane was then probed overnight at 4°C with the respective primary antibodies: NANOG (1:1000, #3580, CST), OCT4 (1:1000, #2750, CST), TDGF1 (1:1000, #2020, CST), ERK1/2 (1:1000, #9102, CST), phospho-ERK1/2 (1:1000, #9101, CST), p38 (1:1000, #Ab7952, Abcam), phospho-p38 (1:1000, #9211, CST), JNK1/2 (1:1000, #9252, Sigma-Aldrich), phospho-JNK1/2 (1:1000, #4668, CST), MEK1/2 (1:1000, #4694, CST), phospho-MEK1/2 (1:1000, #9154, CST) and β-actin (1:10000, #A2228, Sigma-Aldrich). The membrane was subsequently washed in 1X TBST and probed with the following secondary antibodies for two hours: Pierce Goat Anti-Mouse IgG (H+L) Peroxidase Conjugated (1:6000; 1:10000 for β-actin, #31430, Thermo Scientific) and Pierce Goat Anti-Rabbit IgG (H+L) Peroxidase Conjugated (1:6000, #31460, Thermo Scientific). Clarity™ ECL Western Blotting Substrate (Bio-Rad) was used for detection, and the X-ray films were developed using Konica Minolta SRX-101A developer.

### RNA extraction and quantitative reverse transcription PCR

NT2 cells treated with either paraquat, GSH or atRA were lysed in Trizol™ (Invitrogen). The extracted total RNA was quantified by NanoDrop1000. 2 µg of total RNA were treated with the TurboDNase (Ambion) to eliminate residual genomic DNA. The RNA sample was then reverse transcribed into cDNA by using the GoScript Reverse Transcription kit (Promega). The resultant cDNA was used as a template for qPCR on the Fast platform (Applied Biosystems). The relative abundance of gene transcripts was quantified by the –ΔΔCt method. The statistical significance was determined by the Student *t*-test. The primers used for qPCR were as follows: *Cyp26a1* forward primer 5′-gcaggaaatacggcttcatctac-3′ and reverse primer 5′-aggagtcgtgcaggttagagagg-3′; *Gfra1* forward primer 5′-ctgaagcagaagtcgctctacaac-3′ and reverse primer 5′-ggaccacccggaatatatctgac-3′; *Hoxa1* forward primer 5′-acttcactaccaagcagctcacg-3′ and reverse primer 5′-cgaagagctggacttctctgagg-3′; *Keap1* forward primer 5′-tcatccagccctgtcttcaagg-3′ and reverse primer 5′-acatgacagcaccgttcatgac-3′; *Ncam* forward primer 5′-acatcttcagcgacgatagttcc-3′ and reverse primer 5′-ctaattccatggcagtctggttc-3′; *Nestin* forward primer 5′-atctctgggagcatggaacctg-3′ and reverse primer 5′-tcttcccacctctgcacatctg-3′; *Neurod1* forward primer 5′-ctgtccaaaatcgagactctgc-3′ and reverse primer 5′-tctgctcaggcagaaaagtcc-3′; *Nrf2* forward primer 5′-ccagcacatccagtcagaaacc-3′ and reverse primer 5′-agcaatgaagactgggctctc-3′; *Pax6* forward primer 5′-tcagcaccagtgtctaccaacc-3′ and reverse primer 5′-atgcaggagtatgaggaggtctg-3′; *Prdm16* forward primer 5′-accatgtgtcagatcagtgagc-3′ and reverse primer 5′-aagagttcgtcacactcgtcac-3′.
